# Changes in Maternal Leucine-Rich α-2 Glycoprotein Levels During Pregnancy: Comparison Between Preterm and Term Births

**DOI:** 10.7759/cureus.103808

**Published:** 2026-02-17

**Authors:** Takuya Inouye, Mio Kobayashi, Daisuke Higeta, Yoshikazu Kitahara, Akira Iwase

**Affiliations:** 1 Department of Obstetrics and Gynecology, Gunma University Graduate School of Medicine, Maebashi, JPN

**Keywords:** biomarker, inflammation, leucine-rich α-2 glycoprotein, pregnancy, preterm birth

## Abstract

Introduction: The goal of this study was to longitudinally assess the maternal serum leucine-rich α-2 glycoprotein (LRG) level throughout pregnancy and the puerperium, and to explore its association with preterm birth (PTB).

Methods: A total of 37 pregnant women (17 with PTBs and 20 term births as controls) were enrolled in the retrospective study. Serum LRG levels were measured using an enzyme-linked immunosorbent assay at four time points: early, middle, and late pregnancy and puerperium. Placental histopathological examination was performed in selected cases to assess the presence of chorioamnionitis (CAM).

Results: No significant differences in serum LRG levels were observed between the PTB and control groups during pregnancy, and LRG levels remained stable from early to middle pregnancy (p=0.55). In both groups, LRG levels increased significantly during puerperium compared with the corresponding values during pregnancy (p<0.001). Postpartum LRG levels were numerically higher in the PTB group than in the control group (192.5 ± 49.6 μg/mL vs. 163.9 ± 39.1 μg/mL), although the difference was not statistically significant (p=0.057). Among CAM-positive cases, LRG levels tended to be higher in the PTB group than in the controls (266.4 ± 12.8 μg/mL vs. 161.7 μg/mL; p=0.09).

Conclusions: Maternal LRG levels remained stable during pregnancy but increased postpartum, potentially reflecting peripartum inflammation. Maternal serum LRG levels did not differ significantly between preterm and term births. Further studies are required to clarify the significance of puerperal LRG levels.

## Introduction

There are approximately 14.84 million preterm births (PTBs) worldwide each year, with a reported global incidence rate of 10.4% [1]. In Japan, PTB is defined as delivery between 22 and 36 weeks of gestation, with a reported incidence rate of 5.7% [2]. PTB is a major obstetric concern that significantly affects neonatal morbidity and mortality, particularly due to the increased risk of long-term complications, such as cerebral palsy, chronic lung disease, and neurodevelopmental impairment [3]. PTB is a multifactorial condition, with intrauterine infection and infection of fetal adnexal tissues (chorion, amnion, placenta, and amniotic fluid) recognized as the key etiological factors [4]. Previous studies have demonstrated that infection triggers PTB by inducing uterine contractions, promoting cervical ripening, and weakening the fetal membranes [5]. Furthermore, there is evidence that inflammation resulting from infection can negatively affect the fetal environment, contributing not only to PTB, but also to increased risks of acute and chronic neonatal diseases [6]. Therefore, the early detection and appropriate management of infections are essential for the prevention of PTB.

Objective and reproducible biomarkers are indispensable for evaluating infection and inflammation. To date, numerous biomarkers in the maternal serum, amniotic fluid, cervical secretions, and vaginal discharge have been proposed. Representative examples include systemic inflammatory markers such as C-reactive protein (CRP) and white blood cell count, as well as cytokines and proteins such as interleukin-6 (IL-6), tumor necrosis factor-alpha, matrix metalloproteinase-8, and fetal fibronectin [7]. Phosphorylated insulin-like growth factor-binding protein 1, the phosphorylated form of IGFBP-1, has attracted attention as a potential marker of localized inflammation or infection in cervical secretions. However, its clinical utility remains limited [8]. Against this background, the identification of reliable biomarkers that accurately reflect infection and inflammation, and their application in clinical practice, remain important priorities in perinatal medicine.

Leucine-rich α-2 glycoprotein (LRG), a serum protein first described in 1977 [9], is produced by multiple cell types, including macrophages and hepatocytes, and can be quantified using enzyme-linked immunosorbent assay [10]. LRG1 is associated with various diseases such as cancer, diabetes, cardiovascular conditions, and inflammatory disorders, including ulcerative colitis and rheumatoid arthritis [11,12]. It functions as an acute-phase protein, and its levels increase in response to infectious and inflammatory stimuli [12,13]. Owing to these characteristics, LRG has emerged as a potential biomarker of inflammation across multiple fields, including obstetrics. We previously reported that serum LRG levels were significantly higher in patients with ovarian endometriomas than in those with other benign ovarian tumors and that the level decreased following progestin therapy [14]. However, the behavior of LRG during pregnancy is not well understood: there are only a few published studies of LRG in the context of human pregnancy, and the focus of these studies has been conditions such as chorioamnionitis (CAM) and placenta previa [15-17].

The aim of this study was to longitudinally evaluate changes in serum LRG levels throughout pregnancy and puerperium in women who delivered at term, using maternal serum samples collected during gestation and after delivery. In addition, serum LRG levels were measured in women who had PTB to investigate whether the LRG level reflects the inflammatory status during pregnancy and whether it is a potential marker for assessing the risk of PTB.

## Materials and methods

Patients

This study was conducted using maternal serum samples that had been previously collected and stored as part of a prospective observational study at the Department of Obstetrics and Gynecology, Gunma University Hospital. The original study enrolled pregnant women who visited the hospital for prenatal care between January 2019 and December 2022. The control group consisted of women who delivered at term (≥37 weeks of gestation) without underlying medical conditions, such as chronic hypertension, diabetes mellitus, or autoimmune diseases (e.g., systemic lupus erythematosus and antiphospholipid antibody syndrome), and who had no perinatal complications, including hypertensive disorders of pregnancy (HDP), gestational diabetes mellitus (GDM), or placenta previa.

The PTB group included women who delivered at 22­­-36 weeks of gestation. Clinical data, including maternal age, body mass index (BMI), parity, smoking and alcohol consumption, prior miscarriage, gestational age at delivery, birth weight, history of cesarean section, and the presence of perinatal complications, were extracted from medical records.

Perinatal complications included HDP, GDM, and placenta previa. All diagnoses were made based on the definitions provided by the Japan Society of Obstetrics and Gynecology [18]. There are minor differences between the Japanese and international diagnostic criteria; however, the classifications used in this study were based on the Japanese guidelines, reflecting their clinical relevance in domestic practice. The presence of histopathological CAM was also assessed in selected cases based on placental pathology according to Blanc’s classification [19]. Two women in the PTB group who were undergoing steroid treatment for pre-existing conditions were excluded from the analysis.

This study was approved by the Ethics Committee of the Gunma University Hospital (approval numbers HS2018_214 and HS2023_138). As this is a retrospective study using stored data and anonymized samples, informed consent was obtained using an opt-out method through public disclosure on the hospital website.

Serum samples and LRG measurements

Maternal blood samples were collected at four time points: early pregnancy (10-13 weeks), middle pregnancy (25-28 weeks), late pregnancy (34-37 weeks), and puerperium (one to seven days postpartum). Venous blood was centrifuged at 4,000 rpm for 10 minutes, and the resulting serum was stored at −80°C until analysis. Serum LRG levels were measured using a commercial enzyme immunoassay kit (Human LRG Assay Kit; Immuno-Biological Laboratories Co., Ltd., Japan).

Statistical analysis

All statistical analyses were performed using EZR (Saitama Medical Center, Jichi Medical University, Saitama, Japan), a graphical user interface for R (R Foundation for Statistical Computing, Vienna, Austria). EZR is a modified version of R Commander (version 4.2-2), which incorporates statistical functions commonly used in biostatistics.

Between-group comparisons of continuous variables were performed using the student’s t-test, and for categorical variables, the chi-squared test was used. A longitudinal evaluation of serum LRG levels during pregnancy was performed using repeated-measures analysis of variance. To explore the potential confounding effects of mode of delivery, we performed a subgroup analysis restricted to women who underwent cesarean delivery. Statistical significance was set at p<0.05.

## Results

**Figure 1:**
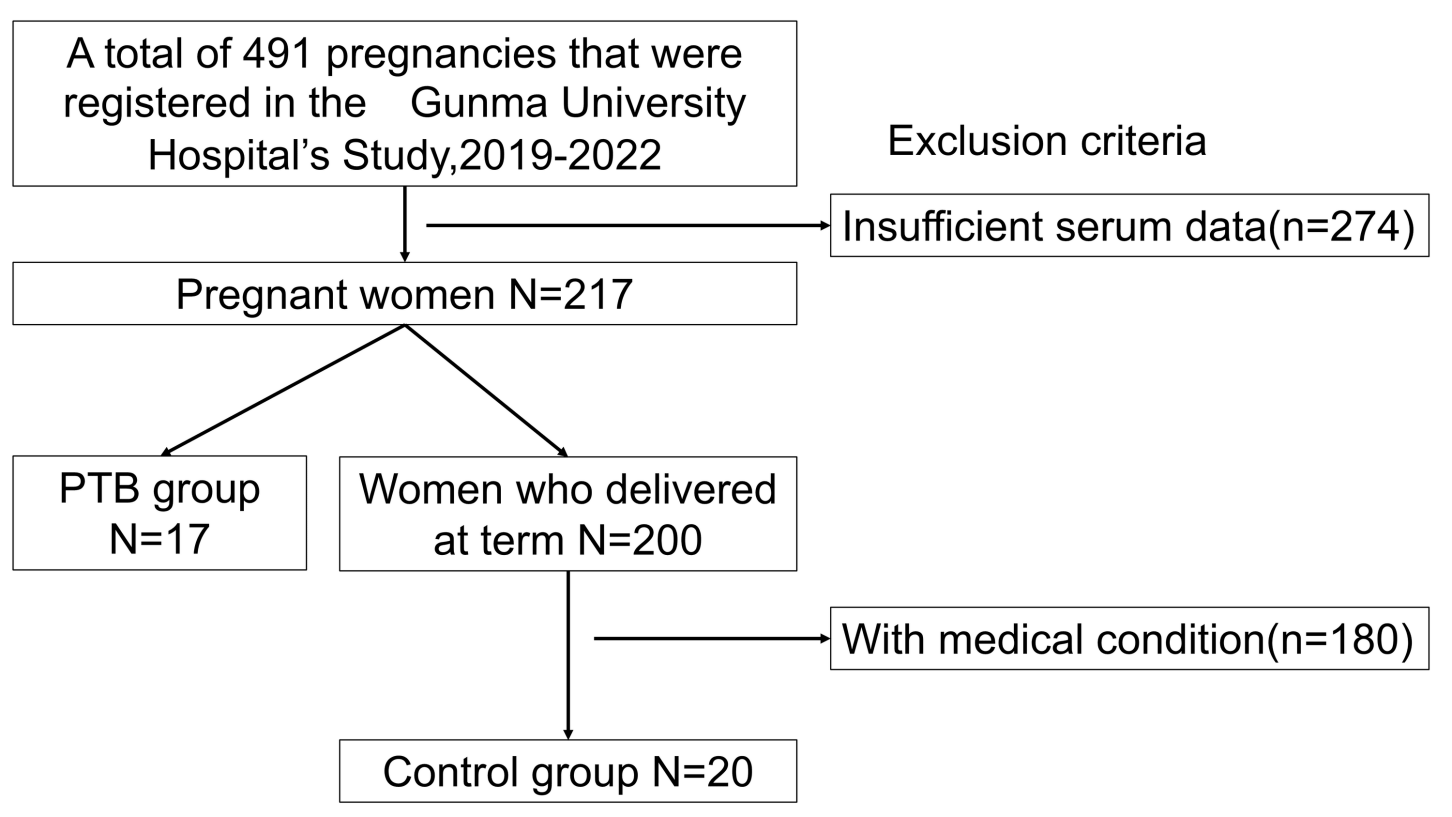
Flow chart of study participant selection Of 491 registered pregnancies, 454 were excluded according to predefined criteria. A total of 37 participants were included in the final analysis, comprising 17 women in the preterm birth (PTB) group and 20 women in the control group.

After applying the exclusion criteria, 37 participants were included in the final analysis: 17 women in the PTB group and 20 women in the control group (Figure 1).

In comparing the two groups, there were no significant differences in any of the baseline characteristics: maternal age, BMI, parity, smoking, alcohol consumption, history of miscarriage, or previous cesarean delivery. In the PTB group, gestational age at delivery was significantly lower, neonatal birth weight was lower, and the rate of cesarean section was significantly higher than those in the control group (Tables 1, 2).

**Table 1:** Baseline characteristics of the study population Values are expressed as mean ± SD. BMI, body mass index; GA, gestational age; PTB, preterm birth; SD, standard deviation.

	PTB (n=17)	Control (n=20)	p-value
BMI (kg/m^2^, mean ± SD）	24.3 ± 5.7	22.0 ± 4.8	0.19
Age (years, mean ± SD）	33.6 ± 4.9	36.3 ± 4.5	0.09
Birth weight (g, mean ± SD）	2155.3 ± 618.2	3094.3 ± 398.0	<0.001
GA at birth (weeks, mean ± SD）	33.8 ± 2.6	38.9 ± 1.2	<0.001

**Table 2:** Baseline characteristics of the study population Values are expressed as a number (percentage). CD, cesarean delivery; PTB, preterm birth.

	PTB (n=17)	Control (n=20)	p-value
Nulliparous (n (%))	5 (29.4)	8 (40)	0.73
Smoking (n (%))	2 (11.8)	1 (5.0)	0.58
Alcohol consumption (n (%))	3 (17.6)	2 (10.0)	0.64
Abortion (n (%))	8 (47.1)	8 (40)	0.75
Prior cesarean section (n (%))	7 (41.2)	5 (25)	0.48
Mode of delivery (CD) (n (%))	16 (94.1)	7 (35.0)	<0.001

Among the total study population, placental histopathological examination was performed in 12 women based on clinical suspicion of CAM. Of these 12 women, histopathological CAM of Grade I or higher was identified in two women in the PTB group and in one woman in the control group.

There were no significant changes in serum LRG levels during pregnancy between early and middle pregnancies within either group (p=0.55) or between the two groups at any gestational stage (Table 3).

**Table 3:** The serum LRG level during pregnancy and the puerperium Values are expressed as mean ± SD (μg/mL). LRG, leucine-rich α-2 glycoprotein; PTB, preterm birth; SD, standard deviation.

	PTB group	Control group	p-value
Early	94.2 (22.6)	93.3 (20.5)	0.89
Middle	91.7 (31.8)	91.9 (30.6)	0.99
Late	-	95.1 (17.7)	-
Post	192.5 (49.6)	163.9 (39.1)	0.057

**Figure 2:**
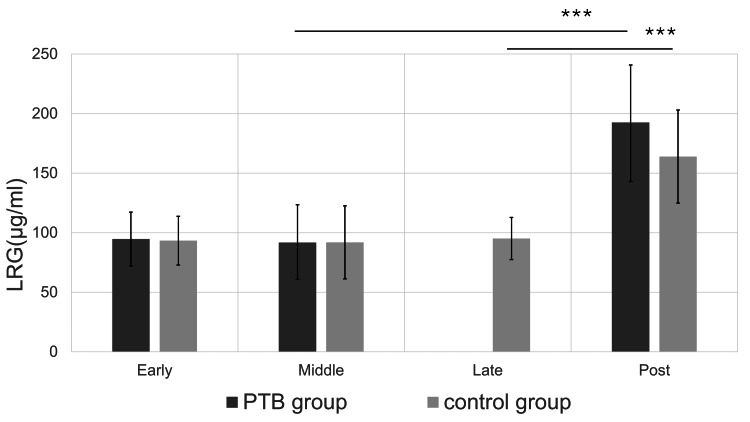
The serum leucine-rich α-2 glycoprotein (LRG) level during pregnancy and the puerperium Mean ± standard deviation values are shown. No significant changes are observed in early or middle-pregnancy between the preterm birth (PTB) and the control group. In the puerperium, the serum LRG level is increased significantly in both groups, compared with the PTB group and the term birth group, respectively (***p<0.001). Late pregnancy data are shown for the control group only, as participants in the PTB group delivered before 36 weeks of gestation and therefore did not have serum samples collected during late pregnancy.

In contrast, both groups had significantly increased LRG levels during puerperium compared with the corresponding values in middle and late pregnancy (Figure 2).

During puerperium, the serum LRG levels were numerically higher in the PTB group; however, the difference was not statistically significant (p=0.057) (Table 3). Among the CAM-positive cases, the serum LRG level was 266.4 ± 12.8 μg/mL in the PTB group and 161.7 μg/mL in the control group, but the difference was not statistically significant (p=0.09). In an additional subgroup analysis restricted to women who underwent cesarean delivery, puerperal serum LRG levels did not differ significantly between the PTB and term control groups (192.4 ± 51.3 μg/mL vs. 186.3 ± 33.9 μg/mL, respectively; p=0.73).

## Discussion

To the best of our knowledge, this study is the first longitudinal evaluation of serum LRG levels during pregnancy. From early to middle pregnancy, no intra-group changes were observed in the PTB or control groups, and there were no significant differences between the two groups. Although the puerperal serum LRG levels tended to be higher in the PTB group, the difference was not statistically significant. Therefore, no definitive conclusion can be drawn regarding the association between puerperal LRG levels and peripartum inflammation. Additionally, although not statistically significant, cases with CAM trended toward an elevated LRG level.

Previous studies have shown that LRG levels increase in cases of fetal infection. Kajimoto et al. reported significantly higher cord blood LRG levels in cases with CAM and fetal infection than in cases with CAM and no fetal infection. Immunohistochemistry of fetal liver tissues from pregnancy termination revealed that LRG expression was localized in hepatocytes in CAM-positive cases, suggesting that the fetal liver is the site of LRG synthesis under inflammatory conditions [15].

LRG has a high molecular weight of approximately 55-60 kDa [20]; consequently, the transplacental passage of LRG is regarded as highly implausible. Thus, the postpartum increase in maternal serum LRG levels observed in this study likely reflects maternal rather than fetal synthesis. Nevertheless, only serum levels were measured in this study; the tissue source of maternal LRG remains unclear.

In this study, the interpretation of puerperal serum LRG levels was complicated by the markedly higher rate of cesarean delivery in the PTB group. Cesarean section involves not only physical stress associated with labor, but also tissue injury related to surgical procedures, both of which are known to induce postoperative inflammatory responses, including increases in inflammatory markers such as CRP and IL-6 [21,22]. Acute-phase proteins, including LRG1, are upregulated in response to inflammatory stimuli. Consistent with this interpretation, an additional subgroup analysis restricted to women who underwent cesarean delivery showed no statistically significant difference in puerperal serum LRG levels between the PTB and term birth groups. However, because of the limited sample size, we were unable to perform more detailed analyses stratified by delivery mode or to examine the associations between LRG and other inflammatory markers, such as CRP or cytokines. Therefore, these findings should be interpreted with caution.

A recent prospective cohort study by Sotodate et al. demonstrated the clinical utility of LRG for the histopathological diagnosis of CAM in cases with PTB [16]. The authors identified umbilical vein LRG level and gestational age as independent predictors of CAM; these factors were shown to have higher diagnostic accuracy than conventional inflammatory markers, such as IL-6 and CRP. Their analysis included 68 PTBs and provided sufficient statistical power to detect significant associations [16]. In contrast, in the present study, there was a trend toward higher maternal serum LRG levels in cases with CAM, but the difference was not statistically significant. This difference may be partly explained by the small number of cases with CAM in our study cohort. Whereas Sotodate et al. measured LRG levels in umbilical vein blood at the time of delivery, in the present study, maternal serum collected between postpartum days one and seven served as the material for analysis. Umbilical vein blood reflects the peak fetal inflammatory response during delivery, whereas the LRG level in the postpartum maternal serum may have already declined. Therefore, differences in sampling sites and timing may have contributed to the weaker associations observed in the present study.

In addition to CAM, elevated maternal LRG levels have been reported in placenta previa, where LRG may serve as a marker of endothelial dysfunction. Ersuz et al. compared third-trimester serum LRG levels among women with placenta previa, those undergoing cesarean section without placental abnormalities, and those with normal vaginal deliveries. The results revealed significantly higher LRG levels in the placenta previa group, suggesting that abnormal trophoblastic invasion may contribute to increased maternal LRG levels [17]. Notably, the absolute maternal serum LRG levels differed from what was found in the present study. This discrepancy may be attributable, at least in part, to the differences in the enzyme-linked immunosorbent assay (ELISA) kits used for LRG measurements, as different assay systems can yield different absolute concentrations. This hypothesis is further supported by the evidence that LRG1 is expressed in endothelial cells. In the kidney, its expression has been demonstrated by in situ co-hybridization with CD31 transcripts and immunohistochemistry of laser-captured glomeruli [23,24]. Similarly, putative LRG1-positive endothelial cells have been identified in the lung and brain tissues [25,26]. Because impaired spiral artery remodeling is also a feature of HDP, maternal LRG levels may also be elevated. Despite this expectation, the present study did not include participants with HDP or placenta previa. This may explain the absence of elevated LRG levels during middle pregnancy.

The results of previous studies have shown that LRG expression is regulated by transforming growth factor-beta (TGF-β) signaling via the endoglin and Smad1/5/8 pathways, particularly under inflammatory conditions [27,28]. Significantly, an elevated TGF-β level in amniotic fluid has been reported in Stage III CAM, suggesting that this pathway contributes to the increase in the postpartum LRG level observed in the present study [29]. On the other hand, the absence of a significant difference in the LRG level between the PTB and control groups during middle pregnancy may reflect insufficient TGF-β activity at that stage.

The limitations of this study should be considered when interpreting the findings. First, the sample size was small, which inherently limited the statistical power. Because this study was conducted at a single center and designed as a retrospective analysis using all available archived maternal serum samples, and because no prior longitudinal studies of maternal serum LRG levels across pregnancy stages and the puerperium existed, an a priori power calculation was not feasible. Accordingly, the lack of statistically significant differences in several analyses may reflect insufficient power rather than the definitive absence of biological effects. Second, the study population was selected. Women with other perinatal complications, such as placenta previa and HDP, were excluded, although maternal LRG levels in these conditions may not be comparable to those in uncomplicated pregnancies. In addition, detailed information on certain clinical factors, such as antenatal steroid administration for fetal lung maturation in PTB cases, was not uniformly available and therefore could not be evaluated in the present analysis. These selection criteria and unmeasured clinical factors may have reduced the heterogeneity of the inflammatory status and limited the external validity of the findings. Third, the interpretation of puerperal LRG levels is strongly influenced by the marked imbalance in the mode of delivery between groups. Cesarean delivery was overwhelmingly more common in the PTB group, and surgical trauma is a well-recognized driver of systemic inflammatory responses. Therefore, the mode of delivery is a major confounding factor. Consistent with this, a subgroup analysis restricted to women who underwent cesarean delivery revealed no statistically significant differences in puerperal LRG levels between the preterm and term birth groups, indicating that the present data do not provide sufficient statistical evidence to support a PTB-specific elevation in LRG. Moreover, puerperal blood sampling occurs over a range of postpartum days (one to seven), during which inflammatory markers may change rapidly. This temporal variability, combined with differences in delivery modes, complicates the interpretation of postpartum LRG levels. Due to the limited sample size, further stratified analyses according to the exact postpartum sampling day were not feasible. Lastly, the study focused exclusively on serum LRG and did not assess other inflammatory mediators or cytokines, such as TGF-β, nor did it include histopathological analyses to localize LRG production in maternal or placental tissues. These limitations restrict the mechanistic interpretation of the findings and underscore the exploratory nature of the present study; however, a key strength of this study is the longitudinal assessment of maternal serum LRG levels from pregnancy through the puerperium using serial measurements.

## Conclusions

In this retrospective study of 27 women who had given birth, maternal serum LRG levels were found to have increased during the puerperium; however, no statistically significant difference was observed between preterm and term birth groups. Given the small sample size and marked imbalance in the mode of delivery, these findings should be interpreted cautiously. Larger studies with adequate control for delivery mode are needed to determine whether maternal LRG differs according to the PTB status.
